# Evidence that hepatitis C virus genome partly controls infection outcome

**DOI:** 10.1111/eva.12151

**Published:** 2014-03-26

**Authors:** Matthew Hartfield, Rowena Bull, Peter A White, Andrew Lloyd, Fabio Luciani, Samuel Alizon, on behalf of the HITS investigators

**Affiliations:** 1 Laboratoire MIVEGEC (UMR CNRS 5290 IRD 224 UM1 UM2)Montpellier Cedex 5, France; 2 Infection and Inflammation Research Centre, School of Medical Sciences, Faculty of Medicine, University of New South WalesSydney, Australia

**Keywords:** hepatitis C virus, *IL28B* SNPs, infection simulation, phylogenetic analysis, trait correlation

## Abstract

Infection by hepatitis C virus (HCV) leads to one of two outcomes; either the infection resolves within approximately 6 months or the virus can persist indefinitely. Host genetics are known to affect the likelihood of clearance or persistence. By contrast, the importance of the virus genotype in determining infection outcome is unknown, as quantifying this effect traditionally requires well-characterized transmission networks, which are rare. Extending phylogenetic approaches previously developed to estimate the virus control over set-point viral load in HIV-1 infections, we simulate inheritance of a binary trait along a phylogenetic tree, use this data to quantify how infection outcomes cluster and ascertain the effect of virus genotype on these. We apply our method to the Hepatitis C Incidence and Transmission Study in prisons (HITS-p) data set from Australia, as this cohort prospectively identified incident cases including viraemic subjects who ultimately clear the virus, thus providing us with a unique collection of sequences from clearing infections. We detect significant correlations between infection outcome and virus distance in the phylogeny for viruses of Genotype 1, with estimates lying at around 67%. No statistically significant estimates were obtained for viruses of Genotype 3a.

## Introduction

The hepatitis C virus (HCV; family *Flaviviridae*, genus *Hepacivirus*) is estimated to infect around 170 million people worldwide, and is a major cause of chronic liver disease (Simmonds 2004). Due to the ensuing health burden caused by HCV, there is a considerable research focus on understanding how the host and the virus genetics shape the infection outcome (Ploss and Dubuisson 2012). There is also a vast array of new therapies being developed that target both host (e.g. cyclophilin inhibitors, microRNA antagomirs (Janssen et al. 2013)) and viral phenotypes (direct-acting antiviral agents targeting the HCV protease and polymerase; Ploss and Dubuisson 2012).

If untreated, acute HCV infections mostly result in one of two infection outcomes. Either the virus is eliminated within 6 months (a ‘cleared’ infection) or it develops into a ‘chronic’ infection that generally persists for life unless cured by antiviral therapy. Spontaneous clearance can occur after 6 months, albeit with a very low probability (much <1% overall; Micallef et al. 2006; Grebely et al. 2012, 2014). Genetic polymorphisms in the promoter region of the host *IL28B* gene that correlate with an increased probability of HCV clearance have been detected (Ge et al. 2009; Thomas et al. 2009; Rauch et al. 2010). It is clear that certain regions of the viral polyprotein are targeted by the host immune responses and are therefore involved in clearance. Specifically, the envelope (*E*) region of the HCV genome harbours a hypervariable region, which is a target for neutralizing antibody response, which in turn has been associated with driving viral evolution (Weiner et al. 1992; Kumar et al. 1993; Fafi-Kremer et al. 2010; Liu et al. 2010). There are also multiple epitopes within the nonstructural (NS) region of the genome that are targeted by cellular immune response (CD8 cytotoxic T cells), giving rise to virus escape variants (Kuntzen et al. 2007; Osburn et al. 2010). These findings argue that an optimal host immune response clears HCV quickly, leading to a progressive purging of virus variants during acute infection. It is currently unclear to what extent strains isolated from cleared or chronic infections differ in their evolutionary dynamics. Farci et al. (2000) found that HCV strains detected during the chronic phase tend to have higher genetic diversity in the E region compared with strains detected in subjects who cleared the infection, leading to clustering of similar infection outcomes along a phylogeny. However, a recent study (Liu et al. 2012) observed the opposite result, with clearing strains exhibiting higher evolutionary rates in the E2-HVR1 region than chronic strains.

Hepatitis C virus is divided into six major genotypes (1–6) and several closely related subtypes (for example, 1a or 1b) that differ approximately by 30% in nucleotide and amino acid sequences (Simmonds et al. 2005). The six genotypes vary in predominance and are dependent upon geographical location (Simmonds et al. 2005). For example, Genotype 1 is most prevalent in the USA and Europe, while Genotype 3 is more common in Asia and South America, and Australia has both genotypes 1 and 3. There also exist epidemiological differences between the genotypes, with Pybus et al. (2001) showing that the 1a and 1b subtypes of HCV spread faster than others.

Overall, it appears likely that specific HCV infection outcomes result from the interlinked effects of the individual virus genome, host genetics and immune response status. However, the relative effects of all of these factors, especially virus genetics, are poorly known. It is hard to investigate the relative impact of each of these effects, especially the impact of genetically similar transmitted viruses, as acute infection is predominantly asymptomatic and the incubation period may be up to several months. Hence, the transmission chain is typically poorly characterized. Recently, phylogenetic methods have been used to determine the effect of a virus genome on the value of infection traits, such as viral load. The power of these methods lies in the fact that a phylogeny built on the sequences from viruses sampled from different individuals can approximate the transmission network between these hosts (Leitner et al. 1996; Hué et al. 2004). Even though this was only an approximation of the real transmission network, Alizon et al. (2010) were able to show using phylogenetic comparative methods that up to 59% of the set-point viral load in HIV infections was attributable to virus genetics effects; this estimate is higher than some estimates obtained from known transmission pairs (Tang et al. 2004; Hollingsworth et al. 2010), but consistent with others (Hecht et al. 2010).

In general, phylogenetic methods have been of considerable use in elucidating the epidemiological history of HCV infections. By estimating the population size of an HCV epidemic in Egypt, it was shown that the outbreak coincided with mass parenteral antischistosomal therapy (Pybus et al. 2003; Drummond et al. 2005; Stadler et al. 2013), validating earlier epidemiological evidence (Frank et al. 2000). Similarly, a phylogenetic analysis of the recombinant HCV form 2k/1b suggests that this variant has spread via infected blood transfusions in the former Soviet Union (Raghwani et al. 2012). However, there is very little work using the phylogenetic comparative approach to determine the genetic effects of the infection itself.

Here, we extend the methods used in Alizon et al. (2010) to HCV, to measure the degree of clustering of discrete infection outcomes along a phylogeny. We describe a method to measure the virus control over the infection outcome for genetically similar HCV strains, which also accounts for phylogenetic and transmission uncertainty. Our virus control value is analogous to heritability in quantitative genetics (Visscher et al. 2008) but applied to categorical traits. That is, the control value measures to what degree a virus genotype influences the probability that a specific infection outcome will be cleared or not, and also that this trait is passed on to its recipient. Our method functions by simulating a set of phylogeny tips (each of which corresponds to an infection outcome) by assuming different virus effects on trait outcomes. We then compare the tip distribution we observe with real data to the simulated tipset, to determine whether there is a virus effect on infection outcome. We additionally describe how the method can take into account confounding effects such as host genetics. These findings have applications in elucidating the causes of an HCV infection outcome, as well as providing evolutionary insights as to the selective forces behind specific infection outcomes.

## Materials and methods

### Ethics statement

Ethical approval was obtained from Human Research Ethics Committees of Justice Health (reference number GEN 31/05), New South Wales Department of Corrective Services (reference number 05/0884) and the University of New South Wales (reference number 05094), all located in Sydney, Australia. The project was approved by the relevant institutional review boards. All subjects provided written informed consent.

### Subjects analysed in study

Hepatitis C virus genome data used in this study were obtained as part of the Australian ‘Hepatitis C Incidence and Transmission Study in prisons (HITS-p) cohort (Dolan et al. 2010; Teutsch et al. 2010). From September 2005 to May 2009, adult prison inmates were recruited at 19 of 30 correctional centres housing adult male or females in New South Wales (NSW). The conditions to join the study were to report a lifetime history of injecting drug use (IDU); to be imprisoned within the last 12 months; and to have a serological test documenting negative anti-HCV antibody status during that time. Of the 500 subjects enrolled in HITS-p, this analysis included subjects who were HCV-RNA negative at enrolment and had completed at least one prospective follow-up visit while in prison (having either been continuously imprisoned or visited upon re-imprisonment after a period of release). A venous blood sample was collected at each interview to screen for HCV antibodies and viraemia (HCV-RNA test), as previously described (Dolan et al. 2010). The average follow-up time between two consecutive interviews was 6 months.

Natural viral clearance was defined as two RNA-negative tests, while antibody-positive status in the subsequent 12 months following the last HCV-RNA-positive test. Within the HITS-p cohort, 134 subjects were found HCV antibody-positive, 94 at baseline and the remaining during the 5 years follow-up period.

Generally, it is hard to obtain virus sequences from subjects who ultimately cleared the infection, as the acute phase of the infection is typically asymptomatic. The HITS cohort therefore provides a unique opportunity for blood sampling in the acute phase, allowing detection of early viraemia in clearing infections. The number of sequences from clearing infections used in this study is therefore one of the highest number to have been analysed. There are only a limited number of publications reporting viral sequences and known disease outcome, including a similar number of clearers (Kasprowicz et al. 2010; Liu et al. 2012). However, these studies have not provided a large cohort of HCV-infected subjects with a closely related transmission network. Rather, these studies match case control samples for specific analyses, which are unsuitable for the measurement of phylogenetic relatedness between viral sequences. The HITS-p cohort is a prospective cohort of individuals infected with the same transmission route in closed setting. Hence, it is likely that the IDUs sampled in this cohort are infected by related viruses, which is though to improve the accuracy of phylogenetic models to approximate the transmission network. HITS-p also collects substantial risk behaviour data on each enrolled individual at each time point, as well as their genotype at the *IL28B* loci.

### Sequences of E1-HVR1

The region encoding the last 171 bp of core, E1 and HVR1 (882 bp; nucleotides 723–1604, with reference to HCV strain H77; GenBank accession number AF009606) was amplified by nested RT-PCR as previously described (Pham et al. 2010). In brief, viral RNA was extracted from 140 μL of plasma using the QIAmp Viral RNA kit according to manufacturers’ instructions (Qiagen, Venlo, Limburg, the Netherlands). Five microlitres of extracted RNA was added to a 15 μL reaction mixture containing 10 μL of RT-PCR reaction mix (iScript One-Step RT-PCR with SYBR Green; Bio-Rad, Hercules, CA, USA), 500 nm of each primer (GV32 and GV33 in Pham et al. (2010)) and 0.4 μL of 50x iScript reverse-transcriptase enzyme (Bio-Rad). Cycling conditions included a 10-min reverse transcription step at 50°C; reverse-transcription inactivation at 95°C for 5 min; 15 cycles at 95°C for 30 s, 55°C for 30 s and 72°C for 1 min, followed by a final extension of 72°C for 7 min. The second-round PCR was prepared by adding 2 μL of first-round product to 18 μL of reaction mix containing iQ SYBR Green Supermix (Bio-Rad) and 500 nm of each primer (GV34 and GV35/GV36). The second-round cycling conditions included 95°C for 5 min and 40 cycles of 95°C for 30 s, 60°C for 30 s and 72°C for 1 min. The amplified products were identified by agarose gel electrophoresis. PCR products were purified and sequenced directly on an ABI 3730 DNA Analyzer (Applied Biosystems, Carlsbad, CA, USA) using dye-terminator chemistry.

### Initial analysis of HCV genetic data

Sequences of the E1-HVR1 region of the HCV genome were available for 92 subjects infected with genotype 1a, 1b or 3a viruses. Of these, 19 were from subjects who ultimately naturally cleared the HCV infection (sequencing was difficult in clearing infections due to lower viral loads). For the study, we only used the earliest-obtained HCV sequence for each patient and discarded subsequent sequences. Preliminary analysis showed that within-host evolution contributed minimally to the phylogenetic relatedness between subjects, as all the longitudinal samples present in the cohort clustered with the subject itself (result not shown). Due to some incomplete sequencing, subsequent phylogenies were built using a 657 bp length of sequence, covering the core and E1 regions. Although Gray et al. (2011) found that this segment was hypervariable within hosts, they also showed that it evolved at a similar rate to the rest of the genome at the between-host level, so phylogenies should not reflect within-host selection at this segment.

**Figure 1:**
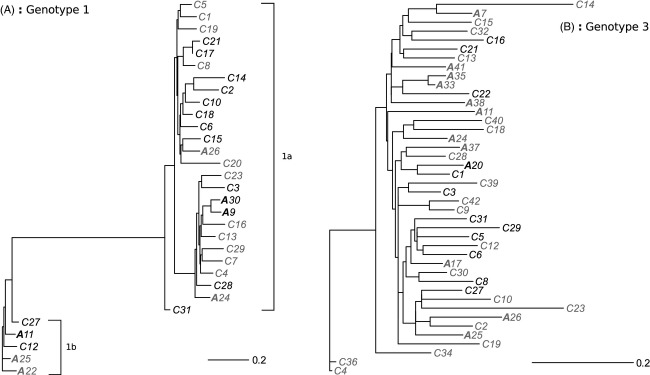
Phylogenies of the two HCV data sets. ‘Genotype 1’ (part A) consisted of viruses of subtype 1a or 1b, as denoted on the graph. ‘Genotype 3’ (part B) consisted of viruses of subtype 3a. Short-term acute outcomes are denoted with an A, chronic outcomes with a C. Grey labels indicate strains where the host had the homozygous *IL28B*-917 SNP corresponding to increased clearance rate. A scale bar is also included that shows the number of substitutions per site. Phylogenies were compiled for this figure using PhyML (Guindon et al. 2010), with a GTR model, gamma-distributed site heterogeneity, empirical nucleotide frequencies and invariable sites. See main text for further details.

We performed an initial analysis on the complete data set. However, to prevent confounding of any detected genetic signal with the HCV genotype, we subsequently extracted the genotypes from the two major observed subtypes and performed analysis on these separate data sets, which we denoted as ‘Genotype 1’ (containing viruses of subtype 1a and 1b) and ‘Genotype 3’ (subtype 3a; Fig. 1). The application of these selection criteria resulted in 31 subjects in the Genotype 1, and 42 subjects in the Genotype 3 category with confirmed sequences amenable for further analyses. We decided to combine sequences of subtype 1a and 1b into the same data set, given their similarity and also because we did not find significant association of acute infections with subtype (*P* = 0.0619, Fisher's exact test).

### Creation of phylogenetic trees

For each data set, we used beast v1.7.3 (Drummond et al. 2012) to create a set of phylogenetic trees for use in our Bayesian analysis, to account for sampling error in the phylogeny. As the inference of phylogenetic signal for a trait can be very sensitive to the underlying phylogeny, then by sampling from the posterior distribution and performing the analysis over several trees, we minimized the influence that phylogenetic sensitivity had on the final result (Parker et al. 2008). The collection date of the HCV sample, as obtained from the HITS-p cohort, was used to align tree tips. Using the jmodeltest software (Guindon and Gascuel 2003; Darriba et al. 2012), it was found that a GTR nucleotide model with estimated base frequencies, invariable sites and a Gamma site heterogeneity model with four categories (GTR + I + Γ) gave the lowest Bayesian information criterion (BIC) score.

We estimated phylogenies by comparing outputs consisting of a constant population size coalescent model; exponential population growth; or a Bayesian Skyline model with 10 groups. Using the Path-O-Gen software (version 1.4, available from http://tree.bio.ed.ac.uk/software/pathogen/), we did not find a positive correlation between the sampling date and root-to-tip divergence for either subtype. Both estimates were not significantly different from zero (correlation coefficient for Genotype 1 was −0.220, *P* = 0.398; for Genotype 3, it was −0.0863, *P* = 0.600). Therefore, it appeared that no molecular clock signal is present in the data. We therefore proceeded using a strict clock model as the simplest possible scenario and checked afterwards that the estimated substitution rates were in line with previous findings for HCV. The outputs seemed to make sense, as substitution rate estimates that we found lied around 10^−3^, as found by Gray et al. (2011). The log-likelihoods of the exponential model and the Bayesian Skyline models were not significantly different when individually compared with the constant-size model, as inferred using a likelihood ratio test. Therefore, we used the outputs from the constant-size coalescent population model for subsequent analyses.

There could be a concern that since we sampled from a densely infected population, then using a coalescent model would provide a skewed phylogeny with branches that are more closely related than they actually are. As a precaution, we also produced phylogenies using a birth–death model, as opposed to a coalescent model (Stadler et al. 2012). We will compare results obtained from different methods where necessary; however, we generally found the same quantitative results as those obtained from a coalescent tree.

We set each MCMC to run for 10^9^ iterations, with parameters logged every 100 000 runs. Each parameter in the MCMC analysis exceeded an effective sampling size of 600.

### Generating simulation data of categorical trait control

In this study, we were interested in estimating how likely it is for a binary trait, such as infection outcome, to be correlated between related virus strains. There currently exists several Bayesian methods to infer genetic relatedness between different tip outcomes from a phylogenetic tree [summarized in Parker et al. (2008)]. While these methods can infer the likelihood of whether a trait is clustered in a phylogeny, they cannot currently quantify the degree to which categorical infection traits are ‘inherited’ by the infection recipient, as well as account for confounding factors. Theoretically, if a trait of interest is more likely to be ‘inherited’ from one infection to the next, then it should cluster around specific tips in the tree. To this end, we constructed a method to simulate inheritance of a trait along the phylogeny, and then link these predicted outcomes to existing statistics that determine the correlation between tip outcomes and phylogenetic distance.

**Figure 2:**
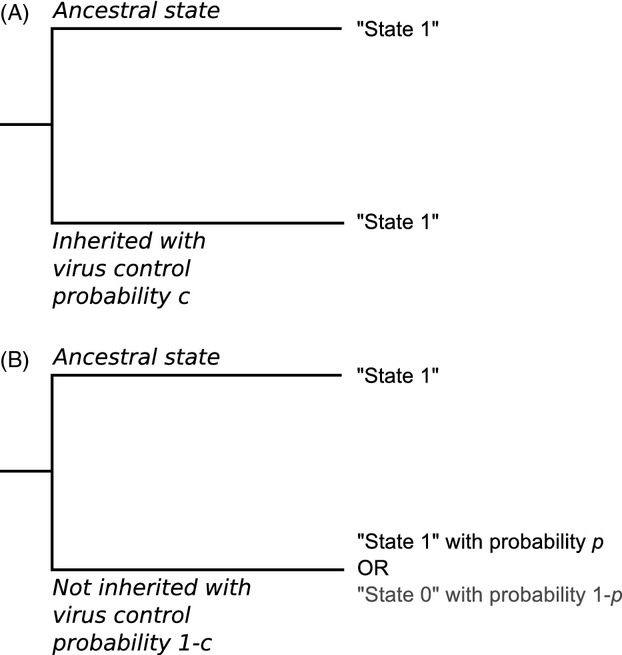
Schematic of the virus control simulations. The root has an initial state assigned to it; this state is certainly passed on to one of the offspring genotypes with probability *c* (case A), or is not passed on with probability 1 – *c* (case B). In this latter case, the offspring state is assigned a value drawn from a Bernoulli trial, where *p* is the observed frequency of the trait of interest from the data set.

A schematic of these simulations is shown in Fig. 2. For the simulations, we chose 500 trees at random from the posterior distribution as produced by the beast analysis for a certain genotype, to make the simulations tractable. For each of these trees, we randomly simulated the trait values for the tree, based on 20 different correlation values (denoted *c*) that were chosen from a uniform distribution between 0 and 1. A value of *c* = 0 means that there is no trait correlation between related strains, so daughter infections are assigned a trait value at random. On the other hand, a value of *c* = 1 means that the ‘offspring’ of an infection caused by a specific strain is certain to retain the same outcome as its ‘parent’ infection. For each of these 20 chosen values of *c*, we started by either assigning the trait of interest (such as HCV infections that clear rapidly) to the root with probability *p*, where *p* is the observed frequency of the trait in the actual data set or the alternative trait (such as chronic HCV infection) with probability 1–*p*. From this tip, we went through each node of the tree in turn. At each new node, we assigned a trait to this node. This trait is either that of its ancestor with control probability *c*. With probability 1 – *c*, the outcome is unrelated to its ancestor, so instead the trait was chosen from a Bernoulli distribution with mean *p* (that was observed in the real data) and subsequently assigned to the node. For each branch, we repeated this process for generations, which were chosen from the following distribution:

g = 1 + Poisson (*L*/*T*_*g*_)

Here, *L* is the length of the branch between a parent and daughter node in years, and *T*_g_ the average length of infection, which was set to 3 years, as based on estimates using surveillance data and molecular sequences (Magiorkinis et al. 2013). Therefore, at least one generation was assumed per branch, along with an estimated number of subsequent transmissions that were not captured by the existing phylogeny (Shirreff et al. 2013). This process was repeated until all nodes had been assigned values, including all tree tips. Simulations were executed using R (R Development Core Team 2008).

After we simulated infection outcomes, we calculated the associated clustering statistics for a simulated outcome. To achieve this, we used the ‘ace’ function, provided as part of the ‘ape’ package (Analyses of Phylogenetics and Evolution) for R (Paradis et al. 2004). This function implements Pagel's maximum-likelihood method (Pagel 1994) for estimating the rate of correlated evolution between several discrete traits along a phylogeny. It does so by estimating the transition probabilities *q*_0,1_ and *q*_1,0_ between the two traits; the probability that a ‘clearer’ genotype will switch to being a chronic genotype, and *vice versa*. There are two probabilities because it can be easier to switch from one state to the other, hence, *q*_0,1_ is not necessarily equal to *q*_1,0_. After estimating these transition probabilities, the inputted control value was saved, along with estimates of the switching rates (with standard errors) and the log-likelihood for the estimates. Note that in order for the analysis to proceed, there needed to be heterogeneity in trait outcomes. That is, there has to be at least one tip of each category present at the end of the simulation, to cause transitions between the two traits. If all the simulated tips were of one type only, we artificially introduced an outcome of the opposite type at a random tip on the phylogeny.

Overall, we ended up with 500 trees × 20 trait control values = 10 000 simulated outcomes for each genotype of interest. Generally, the final outcome corresponded well with actual data; with simulations along the Genotype 1 coalescent phylogeny, the average final number of clearers equalled 6.99, which was not significantly different from the true value of 7 (two-sided *t* = −0.298, *P* = 0.766). The total number of clearers appeared to fluctuate around 7 for most correlation values, but if the correlation value used as an input approached 1, then this total number could reach high values (so all tips would be assigned the infrequent trait), or more likely to go to zero. This behaviour is due to the final trait allocations being more likely to reflect the initial state assigned to the tree root (Fig. S1A). After removing extremely high outlier values (those that lie outside the 97.5% upper quartile), the estimated switching rates were found to be significantly negatively correlated with the input trait correlation values (for *q*_1,0_, *F*_1,9748_ = 135.7, *P* < 2.2 × 10^–16^; for *q*_0,1_, *F*_1,9748_ = 177.4, *P* < 2.2 × 10^–16^; see also Fig. S1B,C). This supported our intuition that with high levels of virus control on trait outcomes, similar outcomes tended to congregate around closely related tips. These results were quantitatively similar for the Genotype 3 data set, and also to results produced using a birth–death phylogeny.

### Using simulation data to quantify virus control over traits

**Figure 3:**
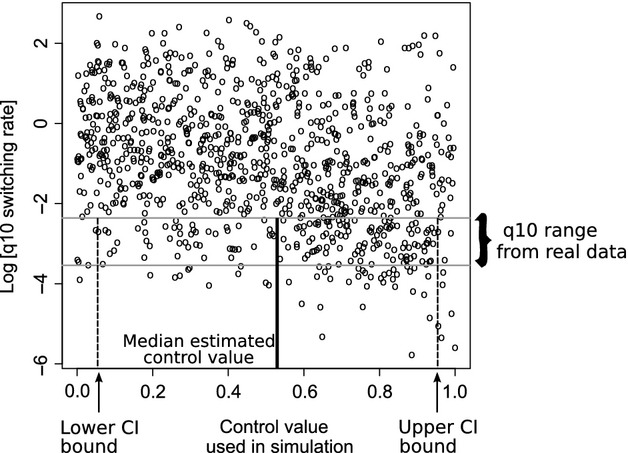
Schematic showing how to estimate virus control *c* from simulation data. After estimating the switching rate (*q*_1,0_) from the true data set (*y*-axis), the simulation points are found that also lie within this range (grey horizontal lines). The associated control values are then found from these points, so the median virus control value is then obtained along with confidence interval limits (black lines on the *x*-axis). Note that for this figure, we only plot a sample of all points on a log scale for clarity of presentation.

To estimate the control of the HCV virus genotype over the infection trait, we estimated the switching rates for the true data set, for 200 trees sampled from the beast analysis. This gave us a range of true switching rates for the actual data. These transition values are important in their own right, as they notify to what degree infection outcomes cluster on the phylogeny. Yet they are different from the main quantity of interest, which is the extent to which the trait is controlled by the virus. Therefore, using these estimates, we then selected simulation data that jointly generated the same switching rates and then obtained the associated correlation values that were used to generate these same values (Fig. 3). This methodology shares many similarities with the Approximate Bayesian Computing (ABC) approach. In both cases, we first simulate many response values (here, the switching rates and log-likelihoods) using many sets of input values (here the virus control values), and then, when we observe such response values in nature, work our way back to infer what the input values are. One difference though is that in ABC the precision has to be set, whereas here we use the quantiles of the real data to generate the confidence intervals for our estimated data.

To test for significance, we randomized the tips 1000 times and obtained the estimated control value for each random tipset. We denoted a control value as being significant if more than 95% of the randomizations were lower than the true value, as randomization should break up associations between tips, thereby decreasing the expected virus control over the trait. The fact that significance was determined using maximum-likelihood estimates of the switching rates meant that these values were optimized based on the underlying phylogenetic tree. Therefore, the randomization procedure tested for clustering between similar traits, while accounting for potential confounding effects of the tree size and shape.

### Testing for virus control over infection outcome while conditioning on host's *IL28B* status

Estimates of infection outcome correlation could have been confounded by the presence of SNPs at the *IL28B* locus that correlate with increased clearance rate. In these cases, we wanted to obtain estimates of the trait correlation while accounting for external factors. To achieve this, we calculated switching rates and log-likelihood values using the bayestraits package (available from http://www.evolution.rdg.ac.uk), which executes the maximum-likelihood analysis of Pagel (1994) while accounting for secondary traits that can affect the evolution of the main trait. We first checked that the same results as the ‘ace’ package for R were obtained for the switching rates if we only investigate one trait, which they did. BayesTraits can thus be used to calculate the estimated transition rates of infection outcome, given that there is a possible covariation with the host's *IL28B* genetic status.

From these estimates, we recalculated the estimated virus control over the trait, given the new transition probabilities, from the simulation data as outlined above. We then tested for significance by randomizing the tips 1000 times, while ensuring that the infection outcome and host *IL28B* factor for a specific tip remain linked together. Trait correlation estimates were then recalculated for each of these 1000 new tip sets and tested for significance as before.

## Results

### Correlation between virus genomes and infection outcome for all genome sequences

**Figure 4:**
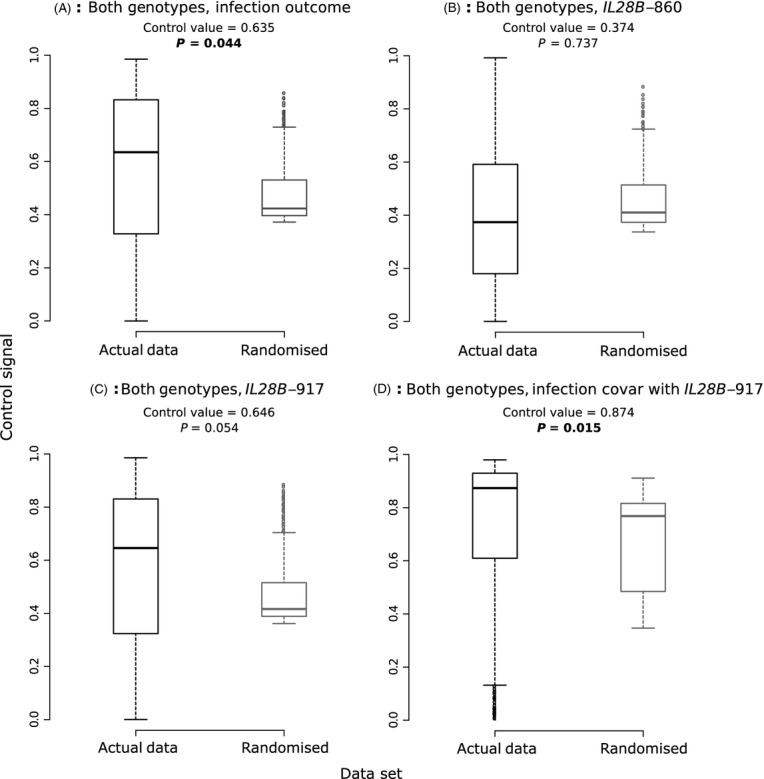
Estimate of control signal for a single trait, based on a coalescent phylogeny for all sequences. Control signal estimates as inferred from the actual data set of interest (black), and of the 1000 median values of virus control estimates obtained from randomized tipsets (grey). *P* values listed above each pair of box plots show significancy of true control value based on randomization test; bold values indicate *P* < 0.05. Data analysed were the infection outcome (A); the status of the *IL28B*-860 SNP (B) or *IL28B*-917 SNP (C); or the the infection outcome covarying with the *IL28B*-917 SNP (D).

Genetic data obtained from the HITS-p cohort mostly resolved itself into one of the two clades, specifically HCV genotypes 1 (including subtypes 1a and 1b), and subtype 3a (Fig. 1). These data sets are labelled as ‘Genotype 1’ and ‘Genotype 3’, respectively. As a first test of our method, we investigated whether we could detect evidence of virus control over infection outcome for both genotypes analysed together (a joint phylogeny is shown in Fig. S2). Results are presented in Fig. 4A, which shows that a fairly high level of virus control was observed (0.63), which was significant (*P* = 0.044 following a randomization test). A lower value (0.59) was observed for the birth–death model, which was also marginally significant (*P* = 0.046; Fig. S3A).

The high level of virus control found for randomized tips likely reflects that, although the log-likelihood of the switching rates decreases with increased virus control, the likelihood surface can be flat for a broad range of control values. This effect can reduce the power of the method to detect low levels of signal (see Fig. S1D for log-likelihood values obtained over the Genotype 1 phylogeny). We will investigate the power further in the section ‘Power calculations and analysis of simulated data’.

For this first analysis, it was assumed that infection outcomes change independently of other factors. This is not quite true, as there are known host polymorphisms that can affect infection outcome. If these host effects cluster on the virus phylogeny, then this host clustering may mask any virus signal that would otherwise be expressed. Therefore, we next investigated the phylogenetic signal of host genetic effects.

### Phylogenetic signal for host's *IL28B* status

It is known that several polymorphisms near the *IL28B* gene in humans correlate with increased clearance rate of acute HCV infection. Those with a homozygous C/C mutation at the rs12979860 SNP (hereafter *IL28B*-860) are much more likely to achieve clearance (Ge et al. 2009; Thomas et al. 2009; Tillmann et al. 2010). Similarly, those with a T/T genotype at the rs8099917 SNP (hereafter *IL28B*-917) are more likely to clear the virus (Rauch et al. 2010).

In theory, as these mutations are present in the host genome and not the virus, and we were analysing virus phylogenies, we should not have detected phylogenetic signal for the host's *IL28B* status. We therefore controlled whether there was evidence of clustering for specific *IL28B* genotypes on the virus phylogeny, as a clustering of host traits could have masked expression of virus control on infection outcome clustering.

Figure 4B,C outlines results when using the SNP that maximized clearance rates for HCV (C/C for *IL28B*-860; T/T for *IL28B*-917) as the trait of interest instead of the chronic/clearer outcomes. No significant correlations were obtained, although *p* values tended to be close to significance for the *IL28B*-917 SNP (Fig. 4C; phylogenetic signal value = 0.646; *P* = 0.054). Results were quantitatively similar if using a birth–death phylogeny (Fig. S3B,C).

The near-significant result for *IL28B*-917 SNP highlights a possibility of nonrandom clustering of host genetic effects on the phylogeny, which could have affected our previous analysis on infection outcome status.

### Correlation in infection outcome, accounting for host status

As there could have been potential confounding effect from the host in determining potential influence of the virus control on infection outcome, we repeated the first analysis while accounting for the host's *IL28B* status. To this end, we used bayestraits (available from http://www.evolution.rdg.ac.uk) to recalculate estimated transitions rates of the infection outcome, given that they could be affected by a secondary trait (in this case, the host's *IL28B*-917 genotype). We did not investigate the effect of *IL28B*-860 SNP as a co-factor as we did not find nearly significant evidence of this trait clustering on any of the phylogenies. This second analysis essentially removed the variance in infection outcomes solely caused by host differences and focused on the virus control on the residual variance. We then used these new rates to calculate estimates of virus control from the simulated data set of infection outcomes.

The results from the repeated analysis are outlined in Fig. 4D. A significant (*P* = 0.015) and high virus control value of 0.87 was obtained if infection outcome covaried with the *IL28B*-917 SNP. The control value obtained using the birth–death phylogeny was slightly lower (0.84) but remained strongly significant (Fig. S3D).

This initial analysis demonstrates how, over our entire data set, there appears to be a strong effect of the virus genotype on infection outcome, especially after correcting for host genotype. However, this analysis does not determine whether this signal is driven by effects of one specific genotype. This could be true if the genotypes respond differently to treatment (Lauer and Walker 2001). In addition, it could be argued that the high level of phylogenetic signal was instead caused by the large phylogenetic distance between the two genotype clades (Fig. S2). There may also be uncertainty about whether the two subclades are from different transmission networks. To this end, we subsequently repeated this analysis but on the separate Genotype 1 and Genotype 3 data sets.

### Separate analysis of genotypes

For this analysis, we first tested whether a model that had different transition rates for each clade provided a better fit than one using the same transition rates for each subclade. Initially, we compared four switching rates (different *q*_0,1_ and *q*_1,0_ values for the Genotype 1 and 3 subclades), as opposed to two rates (same *q*_0,1_ and *q*_1,0_ for each subclade). We found that the four-rate model provided a better fit, as determined by a lower BIC value, irrespective of whether we used a coalescent or a birth–death phylogeny (data not shown). This result reflects the large phylogenetic distance between Genotype 1 and 3 clades (Fig. S2).

**Figure 5:**
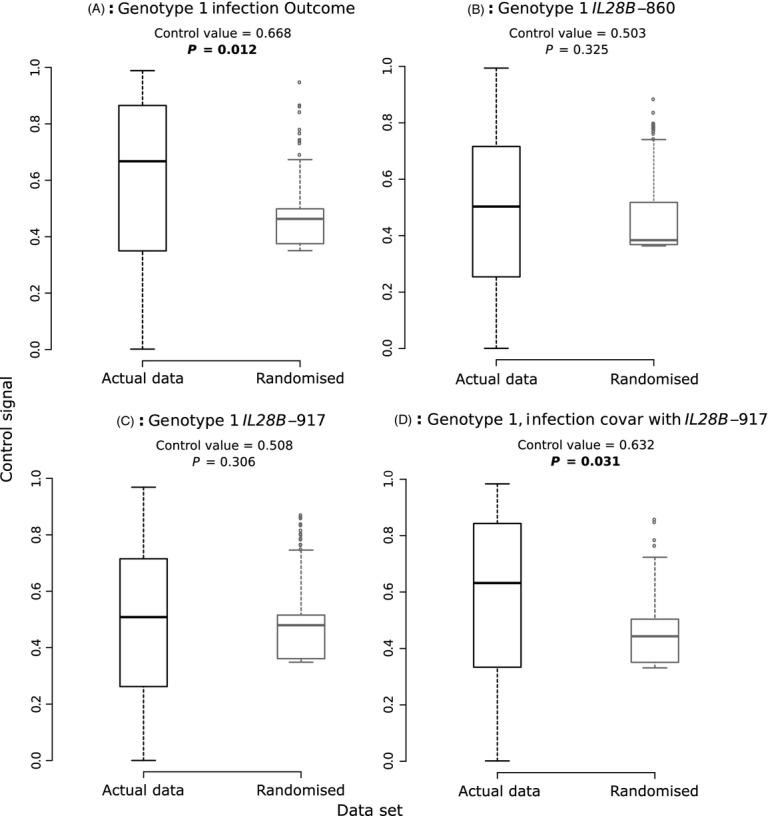
Estimate of control signal for a single trait, based on a coalescent phylogeny, for sequences from Genotype 1. Control signal estimates as inferred from the actual data set of interest (black), and of the 1000 median values of virus control estimates obtained from randomized tipsets (grey). *P* values listed above each pair of box plots show significancy of true control value based on randomization test; bold values indicate *P* < 0.05. Data analysed were the infection outcome (A); the status of the *IL28B*-860 SNP (B) or *IL28B*-917 SNP (C); or the the infection outcome covarying with the *IL28B*-917 SNP (D).

**Figure 6:**
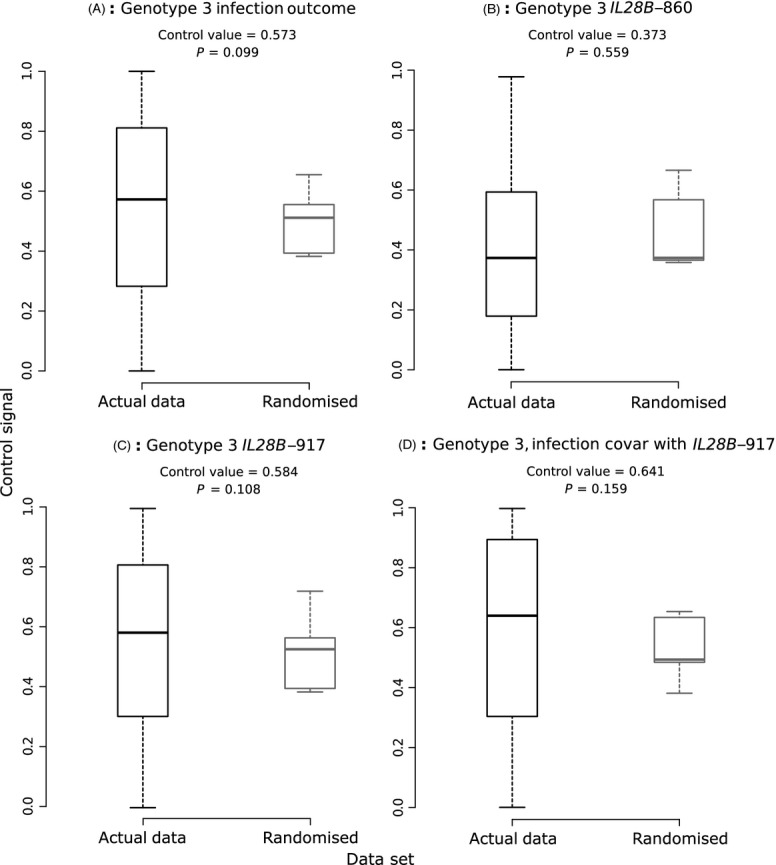
As Fig. 5, but with sequences from Genotype 3.

We next investigated whether we could detect evidence of a significant level of virus control over infection outcome for the two data sets of interest. The results of our analysis are presented for Genotype 1 (Fig. 5A) and Genotype 3 (Fig. 6A). For Genotype 1, a significantly high control value of 0.67 was obtained (*P* = 0.012). However, for Genotype 3, a close but nonsignificant value of 0.573 (*P* = 0.099) was found. Phylogenetic signal for the birth–death phylogeny remained significant for Genotype 1 (Figs S4A and S5A).

It was then determined whether there was any potential clustering of host genotypes along these subclades (Fig. 5B,C for Genotype 1; Fig. 6B,C for Genotype 3). As for the complete data set, we did not obtain any significant results (Fig. 5C; see also Fig. 1 to see clustering of the *IL28B*-917 SNP on the phylogenies).

Note that the degree of observed clustering does not say anything about the correlation between the infection outcome and the *IL28B* SNP. It instead measures whether more related viruses tend to be found in similar host types. One possible reason for signal could be due to selection acting on the hypervariable segment within hosts during infection. To test for this effect, we repeated the analysis with phylogenies built using third codon positions only, which should be largely (but not completely) free from selection. This analysis produced essentially the same estimates of phylogenetic signal as found using a phylogeny built using the whole genetic region (Table S1). Therefore, host clustering does not seem to arise due to selection acting on the hypervariable segment. We will come back to the phenomena of host clustering in further detail in the Discussion.

Finally, we investigated the phylogenetic signal for infection outcome over each subclade, as it covaried with the *IL28B*-917 SNP (Fig. 5D for Genotype 1, 6D for Genotype 3). We found a significant virus control value of 0.63 for Genotype 1; this estimate was not significantly different from the value obtained without correcting for host effects (two-sided *t* = 1.66, *P* = 0.0961). However, despite a higher control value being obtained for Genotype 3 than before (0.64), it was nonsignificant following the randomization test. The estimated control value using the birth–death phylogeny was higher for Genotype 1 (0.69) and remained significant. Although a similarly high value was found for the Genotype 3 birth–death phylogeny (0.66), it was not significant due to the high estimates obtained from randomized tips (Figs S4D and S5D).

Overall, this analysis suggests that the phylogenetic signal obtained for the complete data set was strongly driven by viruses of Genotype 1. After correcting for *IL28B* status, the Genotype 1 estimate was lower than that for the total data set (0.63, compared with 0.87 for all data), implying that the level of virus control could have been inflated when we pooled all samples due to the extra phylogenetic structure caused. We further investigated this result using power simulations, to verify that our estimates for Genotype 1 are accurate.

### Power calculations and analysis of simulated data

In the previous analysis, one of the more glaring outcomes is that even randomized tips gave rather high estimates of virus control, lying at around 40%. The randomization test determined that our data from Genotype 1 showed evidence of phylogenetic signal compared with randomized tips, but the question remained as to whether the magnitude of the estimates of virus control we obtained are meaningful.

To determine the accuracy and the potential power of our method, we simulated 100 different tip outcomes along the Genotype 1 phylogeny, for a known virus control value (*c*), with each outcome produced along a separate tree from the posterior distribution. We then used our method to estimate the level of virus control from these simulated data sets as before, except that we tested for significance using 100 randomized tips as these tests were computationally intensive.

**Figure 7:**
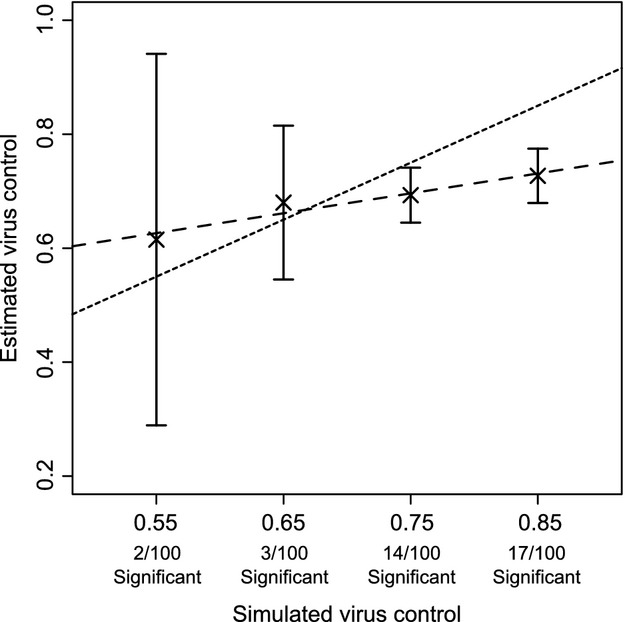
Phylogenetic signal estimated from simulated data with known control value (*c*), along the Genotype 1 phylogeny. 100 tipsets were simulated per input value, with the number of estimates that are significant listed in the *x*-axis. Each point shows the mean value of the significant estimates, with error bars representing 95% confidence intervals. The dotted line shows the *y* = *x* line; the dashed line shows a linear regression fit, with slope 0.35 (adjusted *R*^2^ = 0.88, *P* = 0.0392).

Figure 7 shows the results of this analysis. We see that as the level of simulated virus control *c* increases, the number of significant runs, and thus the power of the method, also increases. However, the power is generally quite low (ranging from 2/100 runs being significant if simulated *c* equals 0.55, to 17/100 if *c* = 0.85). In addition, the estimated level of virus control from the significant estimates increases with the input value (the slope of the linear fit is 0.35). Although error estimates are quite large for inputted *c* = 0.55, we see that the estimated values are fairly accurate in estimating the actual input by comparing mean points to the *y* = *x* line. However, if the input value is very large, then the control value is underestimated (when we set *c* = 0.85, the estimated level of phylogenetic signal is 0.73).

Overall, these power simulations showed that given a result is significant, the obtained estimate of virus control is likely to lie close to the actual level of phylogenetic signal that is present. However, this method also shows that our method can be prone to noisy estimates, which reduced the power of the analysis. This result makes clear the need for future work to increase the precision of phylogenetic inference (Shirreff et al. 2013).

## Discussion

While there are known host factors that can determine the infection outcome of an HCV infection (Ge et al. 2009; Thomas et al. 2009; Rauch et al. 2010; Tillmann et al. 2010), there currently exists very little information on how virus genetics affect the infection outcome. Using a phylogenetic method combined with data from an Australian prisoner cohort, we showed that diversity in the virus genotype could partly explain diversity in the infection outcome.

In our initial analysis, we detected a high level of phylogenetic signal once correcting for the host's *IL28B*-917 SNP, indicating that a virus genomic effect could be present (Fig. 4). We then analysed each subclade separately as the high signal level could have been caused by general differences between genotypes. When investigating only the phylogenetic distance correlation between virus sequences and infection outcomes, we found a significant signal for Genotype 1 (Fig. 5A) but not for Genotype 3 (albeit our result was close to significance; Fig. 6A). We then repeated this analysis while accounting for the host's *IL28B* genotype as a co-factor to explain non-*IL28B* clearance effects and found that the virus control value equalled 0.63 for Genotype 1 (Fig. 5D), which was not statistically different to the estimate without the correction (0.68).

Therefore, our analyses suggest that for Genotype 1 of HCV, a substantial component of clearance that is not due to the *IL28B* genotype is likely to be explained by the similarity of viral sequences infecting the host. However, we did not detect a significant effect for Genotype 3. The most parsimonious explanation for not detecting signal is probably that it is too weak to be picked up by the current method: our power calculations suggest that the signal has to lie at about 50% to be detected (see also Shirreff et al. (2013)). In addition, the coalescent effective population size is much higher for Genotype 3 (∼223 for Genotype 1, compared to ∼1398 for Genotype 3), suggesting that this clade has been undersampled so as to lose phylogenetic signal. The addition of more sequences could therefore help to detect significant signal for Genotype 3.

While the clustering of infection outcomes suggests that there is an effect of virus genetics on infection outcome for Genotype 1, caution should be taken in interpreting the magnitude of these estimates as phylogenetic signal could also arise due to the action of an unknown co-variate that has an effect on virus persistence, such as age or gender. Additionally, it could be argued that infection outcomes cluster due the effect of a different disease that is co-transmitted with HCV. With the HITS-p cohort, the overwhelming majority of transmissions are caused via contaminated injecting or tattooing equipment (Dolan et al. 2010; Teutsch et al. 2010), so co-transmission of other pathogens is unlikely. This is because other blood-borne pathogens that could be transmitted, notably HIV and Hepatitis B, are present at low frequencies in the prisons (<1% for HIV and 5% for HBV). Furthermore, entry into the cohort is only permissible to those not already infected by HIV, so outcome clustering is not due to the effect of HIV co-transmission.

### Host clustering on a virus phylogeny

An important discussion point is why we detected relatively high and near-significant rates of correlation amongst the host *IL28B* genotypes when we pooled all the data, as theoretically there should not have been any magnitude of host genetic outcomes clustering along the phylogeny, which was built based using virus genotypes only. One explanation is that host clustering arose due to a quirk of host sampling, if hosts were genetically related (for example, if they were twins) and also shared the same transmission history. In this regard, no known first-degree relatives were enrolled in the cohort, so this type of sampling bias should not exist.

A more exciting explanation is that there was an interaction between the lineage of the virus and the host genetics. This effect could have arisen if the host acts as a virus filter, so only some HCV viruses can infect hosts that have *IL28B* genotypes that increase clearance rate. Another explanation is that the host *IL28B* genotype selects for specific virus mutations from amongst the quasispecies shortly after the infection has occurred, explaining their genetic similarity. This would be a case of parallel evolution (organisms that have evolved in similar environmental conditions and face the same challenges tend to look alike). However, as phylogenetic signal was nearly significant for only one host SNP (*IL28B*–917) for the complete data set only, and the same degree of signal was also present on a third-site phylogeny (where most selective effects of the virus should be absent), then these findings do not strongly suggest that there is major selection acting by the host. Importantly, this significant interaction we detected between the virus phylogeny and the host genetics should not be mixed with the known correlation between host genetics and infection outcome (Thomas et al. 2009). However, one interesting extension would be to determine the effect of the virus phylogeny on inferring the correlation between host *IL28B* polymorphism and infection outcome. This has been done for HIV (Bhattacharya et al. 2007), but to achieve this for HCV, a larger data set is required.

### Perspectives

It is becoming increasingly recognized that the variance in viral infection outcomes is not just due to host genetics; differences in the virus also plays an important role (Alizon et al. 2011). While there is now substantial work investigating how infection outcomes correlate amongst related strains in HIV [reviewed in Müller et al. (2011)], little work has been undertaken for other viruses of humans, including HCV. The results of this study show that there is a correlation in HCV infection outcome between related strains, for Genotype 1 at least. These findings have important implications in the prognosis of HCV infections in general and can be used to improve and potentially generate new virus-specific or host-specific therapies.

These findings also shed some interesting light on the process of virus evolution. Superficially, it seems baffling as to why HCV infections that clear immediately after the acute stage should be maintained by the virus genotype, as their presence appears to reduce opportunities to spread the pathogen. It could be that ‘clearer’ HCV genotypes are weakly deleterious, as reflected in the low frequency of clearers that are observed. These weakly deleterious genotypes could be maintained between hosts at low frequencies via recurrent mutation (Wright 1931), which could lead to clearer HCV infections having similar genotypes, if mutation is required at specific sites to cause a clearing infection. This mechanism could also be an example of ‘short-sighted’ evolution of HCV, in which clearing outcomes evolve due to a within-host benefit, but at a cost of reduced transmission (Levin and Bull 1994). We therefore anticipate that these results will motivate further work into elucidating the interactions between virus and host genotypes, and also into how these interactions affect the evolution of virulence and pathogen transmission. A phylogenetic analysis of ancestral ‘founding’ variants could be used to investigate this hypothesis; if clearing variants were deleterious in the long term, then they would become extinct over short timescales. Otherwise, they would persist and form distinct subclades.

Our method is able to elucidate correlations between virus genetics and categorical infection outcomes that may not be detectable using standard clustering statistics (Parker et al. 2008). In contrast with earlier methods, this method also provides a quantitative estimate of virus control over a categorical trait, as well as taking into account uncertainty in phylogenetic sampling, which is a common issue (Parker et al. 2008). However, the method can be inherently noisy, as reflected in the high signal levels detected for randomized tips and the fact that we did not detect significant signal for Genotype 3, even after correcting for the host's genotype (see also power simulations, Fig. 7). We anticipate that if more sequences were available, then the method would be less affected by its inherent noise. Secondly, phylogenies were constructed based on genetic data of only a part of the HCV genome, as opposed to whole-genome sequences. Analysing longer sequences or different regions of the HCV genome might result in phylogenies that more accurately reflect the complete set of virus variants. Some areas of the genome have minimal host interactions, whereas other regions strongly interact with the innate and adaptive elements of the host immune response. Therefore, phylogenies based on other parts of the virus genome could also reveal different interaction effects with host genetics. Finally, we hope that future research would refine methodology, so that it would be more likely to detect low levels of phylogenetic signal.

## Conclusion

In summary, by creating a method to simulate inheritance of a binary trait along a set of phylogenetic trees, we have found that the HCV phylogeny controls 67% of the phylogenetic signal of acute HCV infection for Genotype 1. This outcome could have arisen due to an inherited or transmitted trait within the HCV genome. Overall, this finding not only suggests that the virus genotype can affect infection outcome, but this is also dependent on the specific subtype of HCV, and host genetics. These findings should motivate further research into host–parasite interactions that affect virus evolution.

## Data archiving statement

HCV genome samples have been uploaded to GenBank (accession IDs KJ437271 – KJ437343). Simulation data used for analysis are available from the Dryad Digital Repository: http://dx.doi.org/10.5061/dryad.3978f.

## Supporting Information

**Table S1.** Overview of third-site only phylogeny results.

**Figure S1.** Plots of how various simulation statistics vary with increased virus control for our HCV ‘Genotype 1’ coalescent phylogeny.

**Figure S2.** Phylogeny of the joint Genotypes 1 and 3 data set.

**Figure S3.** Estimate of control signal for a single trait, based on a birthdeath phylogeny for all sequences.

**Figure S4.** Estimate of control signal for a single trait, based on a birthdeath phylogeny, for sequences from Genotype 1.

**Figure S5.** As Fig. S4, but with sequences from Genotype 3.
