# Supplementary material for: Evidence that hepatitis C virus genome partly controls infection outcome
Source: Evol Appl. 2014 Mar 26;7(5):533–47. doi: 10.1111/eva.12151 (PMC4055175; doi:10.1111/eva.12151)
Supplement: Supplementary file 1 [file eva0007-0533-SD1.pdf]

Table S.1: Overview of third-site only phylogeny results.

Phylogeny Type	Tipset	3rd Site control value	Full data control value
Constant	Gen. 1 *IL28B*-917	0.504	0.508
	Gen. 3 *IL28B*-917	0.577	0.584
Birth-Death	Gen. 1 *IL28B*-917	0.434	0.448
	Gen. 3 *IL28B*-917	0.575	0.557

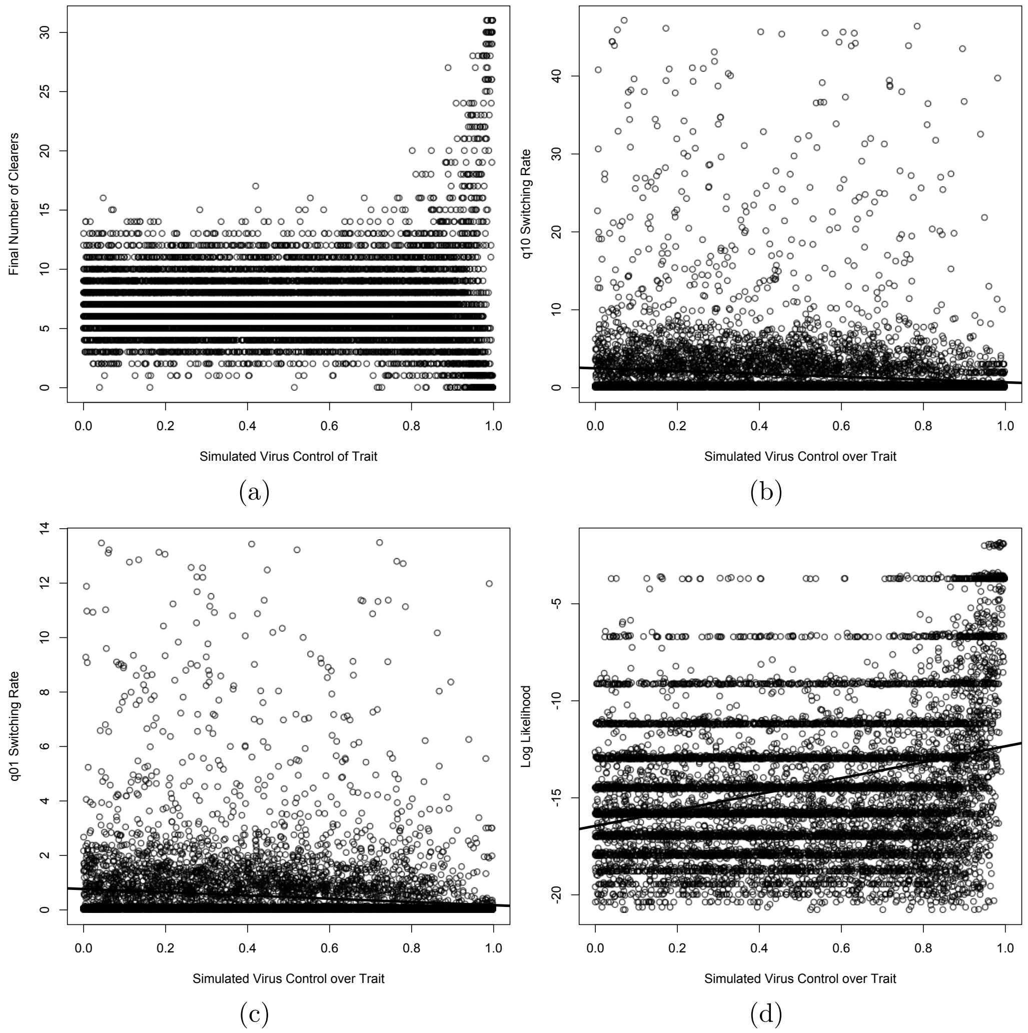

Figure S.1: **Plots of how various simulation statistics vary with increased virus control for our HCV 'Genotype 1' coalescent phylogeny.** (a) Final number of clearers at the tips in the simulated datasets, as a function of the virus control of the trait between related virus sequences. (b) Switching rate from the clearer to chronic outcomes along the phylogeny, as estimated using the 'ace' function, after removing outliers (the highest 2.5 % of values). The line shows the least-squares regression fit. (c) Same as (b) but for the chronic to clearer switching rate. (d) Log-Likelihood of 'ace' analysis. The line shows the least-squares regression fit.

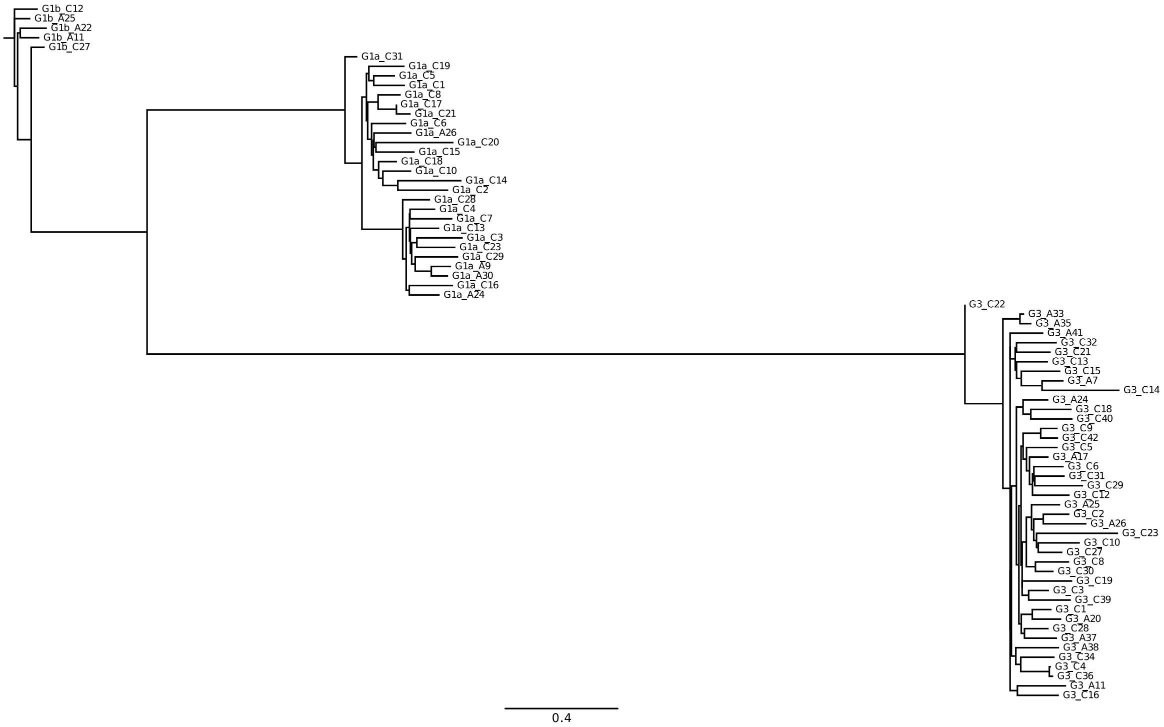

Figure S.2: **Phylogeny of the joint Genotypes 1 and 3 dataset.** Tip labels starting with ‘G1a/b’ are from the genotype 1 a or b dataset respectively, whilst ‘G3’ are from Genotype 3. Short-term acute outcomes are denoted with an A, chronic outcomes with a C. A scale bar is also included that shows the number of substitutions per site. Phylogenies were compiled for this figure using PhyML (Guindon et al. 2010), with a GTR model, gamma-distributed site heterogeneity, empirical nucleotide frequencies and invariable sites. Figure was produced using the FigTree software (`http://tree.bio.ed.ac.uk/software/figtree/`).

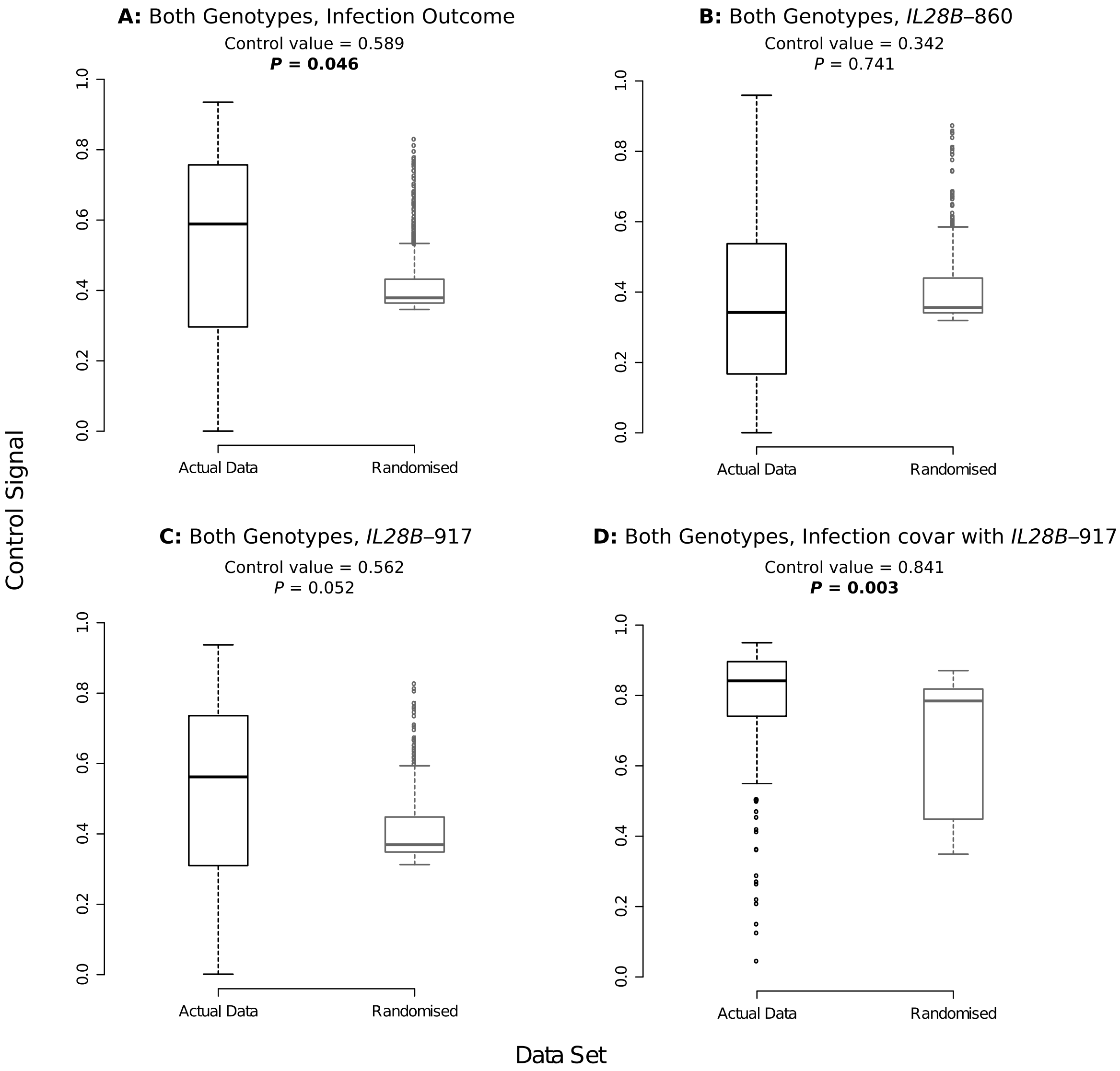

Figure S.3: **Estimate of control signal for a single trait, based on a birth-death phylogeny for all sequences.** Control signal estimates as inferred from the actual dataset of interest (black), and of the 1,000 median values of virus control estimates obtained from randomised tipsets (grey). P values listed above each pair of box-plots show significancy of true control value based on randomisation test; bold values indicate $P < 0.05$. Data analysed was the infection outcome (A); the status of the *IL28B*-860 SNP (B) or *IL28B*-917 SNP (C); or the the infection outcome covarying with the *IL28B*-917 SNP (D).

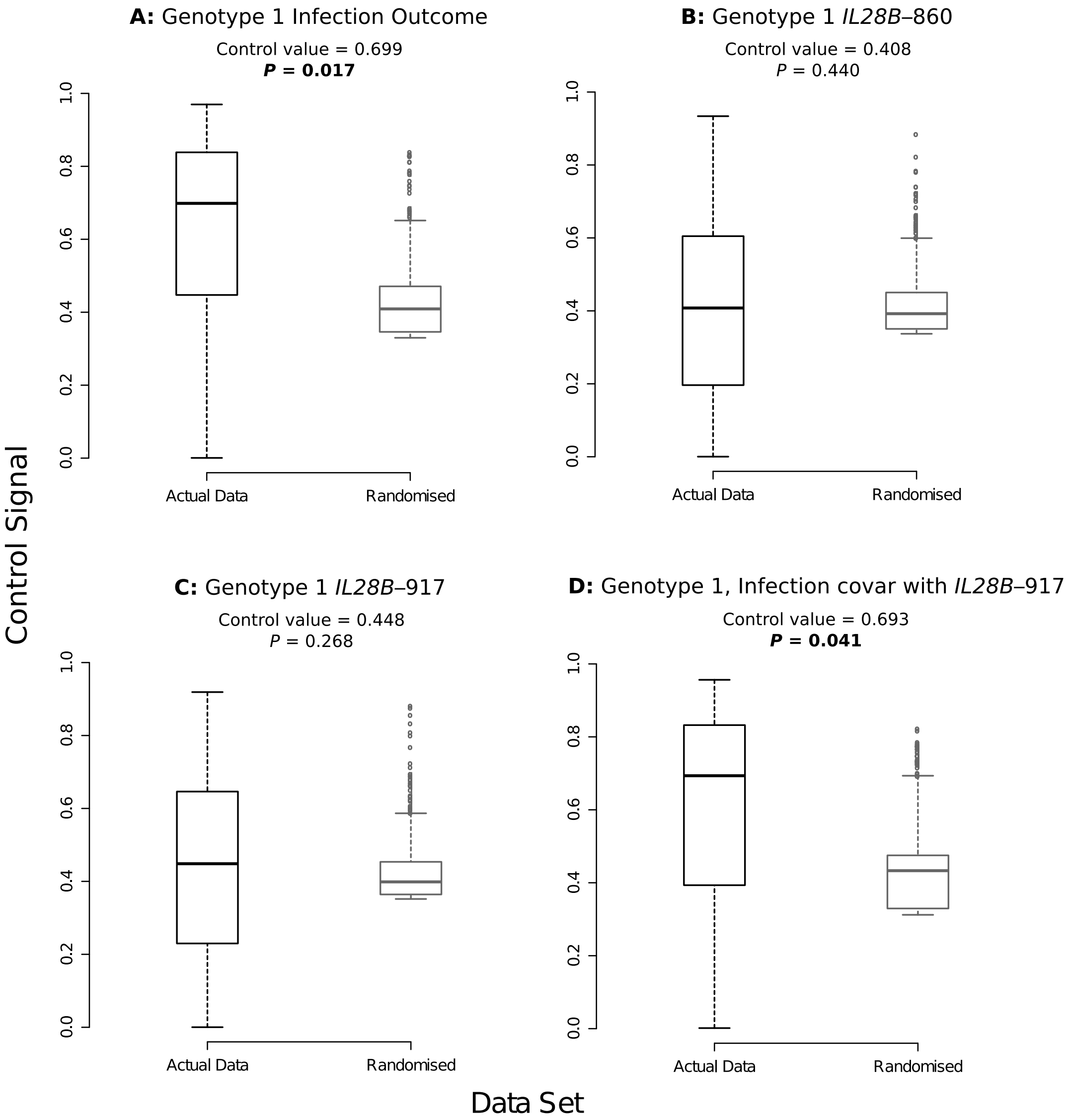

Figure S.4: **Estimate of control signal for a single trait, based on a birth-death phylogeny, for sequences from Genotype 1.** Control signal estimates as inferred from the actual dataset of interest (black), and of the 1,000 median values of virus control estimates obtained from randomised tipsets (grey). P values listed above each pair of box-plots show significancy of true control value based on randomisation test; bold values indicate $P < 0.05$. Data analysed was the infection outcome (A); the status of the *IL28B*-860 SNP (B) or *IL28B*-917 SNP (C); or the the infection outcome covarying with the *IL28B*-917 SNP (D).

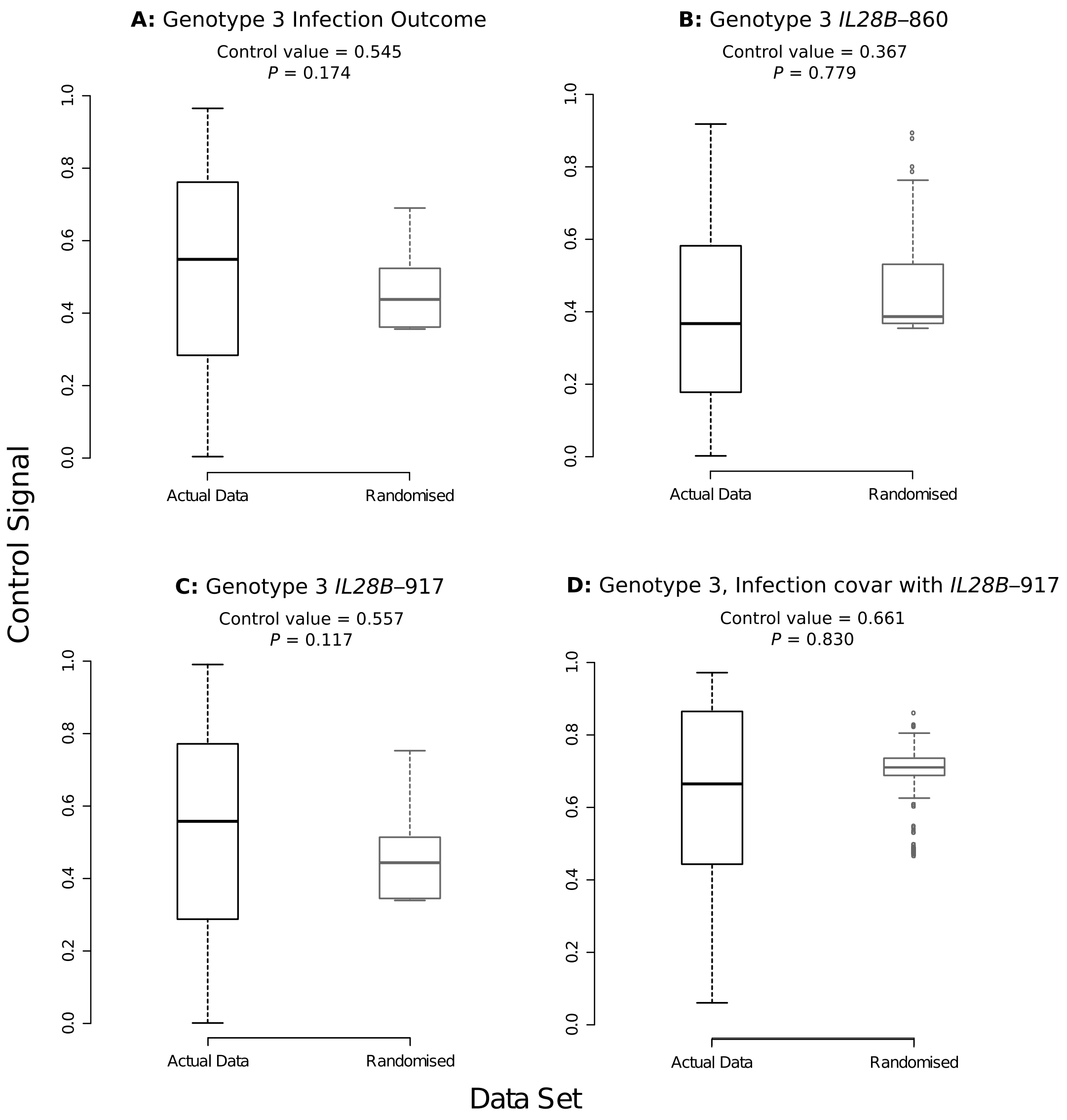

Figure S.5: **As Figure S.4, but with sequences from Genotype 3.**
